# Efficacy of an imidacloprid/flumethrin collar against fleas, ticks, mites and lice on dogs

**DOI:** 10.1186/1756-3305-5-102

**Published:** 2012-05-30

**Authors:** Dorothee Stanneck, Eva M Kruedewagen, Josephus J Fourie, Ivan G Horak, Wendell Davis, Klemens J Krieger

**Affiliations:** 1 Bayer HealthCare AG, Animal Health Division, 51368, Leverkusen, Germany; 2 ClinVet International, 11186, Universitas, 9321, South Africa; 3 Department of Zoology and Entomology, University of the Free State, Bloemfontein, 9301, South Africa; 4 Department of Veterinary Tropical Diseases, Faculty of Veterinary Science, University of Pretoria, Onderstepoort, 0110, South Africa; 5 Bayer HealthCare LLC, Animal Health, 12809 Shawnee Mission Parkway, Shawnee, KS, 66216, USA

**Keywords:** Imidacloprid, Flumethrin, Efficacy, Safety, Fleas, Ticks, Lice, Mites, Dogs

## Abstract

**Background:**

The studies reported here were conducted to ascertain the efficacy of imidacloprid/flumethrin incorporated in a slow-release matrix collar, against infestations of dogs by fleas, ticks, mites and lice. Efficacy was evaluated against the flea *Ctenocephalides felis felis*, the ticks *Rhipicephalus sanguineus*, *Ixodes ricinus*, *Ixodes scapularis*, *Dermacentor reticulatus* and *Dermacentor variabilis,* the mite *Sarcoptes scabiei* and the biting louse *Trichodectes canis*.

**Methods:**

Groups of collar-treated dogs (n = 7–10) were infested with fleas and/or ticks at monthly intervals at least, over a period of up to 8 months. Efficacy against fleas was evaluated 24 to 48 h after treatment and 24 h after each re-infestation. Efficacy against ticks was evaluated at 48 h (acaricidal), 6 h (repellent) and 48 h (sustained) after infestation. The effect of regular shampooing or immersion in water on the efficacy of the collars was also tested. Efficacy against flea larvae was assessed by incubating blanket samples after dog contact with viable flea eggs. Effectiveness against lice and mites was evaluated after treatment of naturally infested animals. With the exception of the mites, efficacy was calculated by comparison with untreated negative control groups.

**Results:**

Efficacy against fleas (24 h) generally exceeded 95%, and against flea larvae it exceeded 99% for 8 months. Sustained acaricidal (48 h) efficacy, covering a period of 8 months was 100% against *I. ricinus*, starting 2 days after treatment (*in vivo*), and 100% against *I. scapularis* (*in vitro*), above 97% against *R. sanguineus,* generally above 97% against *D. reticulatus* and above 90% for *D. variabilis*.

Repellent (6 h) efficacy 2 days after treatment and continuing for 8 months was consistently 100% against *I. ricinus*, and above 90% against *R. sanguineus*.

Regular shampooing affected efficacy against fleas and ticks to a lesser extent than regular immersion in water.

The collars eliminated *Trichodectes canis* within 2 days and *Sarcoptes scabiei* within 3 months.

**Conclusion:**

The rapid insecticidal and acaricidal properties of the medicated collars against newly-acquired infestations of fleas and ticks and their sustained high levels of preventive efficacy have been clearly shown. Consequently they have the potential to prevent the transmission of vector-borne diseases and other conditions directly associated with infestation throughout an entire season of parasite abundance.

## Introduction

Fleas, ticks, and lice belong to the most well-known groups of ectoparasites that infest humans and their domestic animals. It can readily be assumed that from the earliest times they were detested because of the irritation associated with their bites or attachment. However, it is only fairly recently that their roles as vectors of a wide range of viral, bacterial and protozoan diseases and also of helminths affecting man and his domestic animals have been unravelled [1-3]. As a consequence they have now become a major focus of medical and veterinary research and their control an ongoing battle.

### Fleas

It would seem that fleas belonging to the genus *Ctenocephalides* had their origins in Africa (Ménier *et al*. [4] and Beaucournu *et al.*[5] recognize 13 species in this genus). Undoubtedly the most important of these, *C. felis*, has two subspecies, namely *C. felis felis* and *C. felis strongylus*. The so-called cat flea *C. felis felis* feeds on domestic cats and dogs, and has been transported worldwide by humans on their pets, and is seldom if ever found on wild felids [6]. However, its sibling subspecies, *C. felis strongylus* has largely remained a parasite of small wild felids in Africa [5,6]. *C. felis felis* is often more frequently encountered on dogs than the so-called dog flea, *C. canis*. In addition to the irritation caused by fleas, dogs often develop a condition known as flea allergy dermatitis [7] resulting from repeated exposure to flea bites. Furthermore, *C. felis felis* is an intermediate host of the tapeworm *Dipylidium caninum*[1].

The eggs and the three larval stages of dog and cat fleas as well as the pupa are free-living within the sleeping and resting environment of dogs, while the adults are for all practical purposes permanent parasites on their hosts [8]. Under conditions of optimal temperature and humidity, the life cycle from egg-laying until the emergence of adult fleas from pupae, can be completed in 23 days [8].

### Ticks

Ticks act as vectors of a wide variety of protozoan, bacterial and viral diseases in domestic livestock and companion animals [3,9]. There are four stages in the life cycle of ixodid ticks, namely eggs, larvae, nymphs and adults. Once the eggs have hatched, all subsequent stages require a blood meal before they can continue their development.

Several surveys on the ticks infesting dogs have been conducted in various European countries and *Dermacentor reticulatusIxodes ricinusIxodes hexagonus* and *Rhipicephalus sanguineus* were commonly recovered [10-12]. In Britain and Ireland *I. ricinus* followed by *I. hexagonus* were the tick species most prevalent on dogs [13-15], and no *R. sanguineus* was recovered in any of the British surveys.

In studies conducted in the southern USA, *Dermacentor variabilisIxodes scapularis* and *R. sanguineus* were common parasites of dogs [16,17], while the only *Rhipicephalus* species present in South America is *R. sanguineus*, and it was introduced by humans [18]. In surveys in southern Africa and eastern Australia *R. sanguineus* was the dominant species [19-23]. It would thus appear that depending on locality, *I. ricinusI. scapularis D. reticulatusD. variabilis* and *R. sanguineus* are amongst the tick species most frequently encountered on dogs in both Hemispheres.

### Mites

The mite *Sarcoptes scabiei* is the cause of sarcoptic mange or scabies in a wide range of host species. By preference the mites colonize the less hairy regions of the host’s body. Clinical signs in dogs include a fairly constant pruritus, an erythematous rash, and papules and yellowish crusts that form on the skin surface as well as alopecia [24].

### Lice

While infestation with fleas and ticks may be common, infestations with lice are usually rare on dogs, and are often associated with dogs debilitated by disease, age or neglect, on which burdens may become exceedingly large [25].

### Active ingredients

Imidacloprid is an ectoparasiticide belonging to the group of chloronicotinyl compounds, and has been registered in European Union (EU) member states as a veterinary medicinal product for use on dogs and cats since 1997. Flumethrin is an ectoparasiticide that belongs to the group of α-cyano-pyrethroids, and has been registered in EU member states for use on companion and food-producing animals since 1986. These chemicals have been combined in a slow release matrix collar formulation incorporating 10% imidacloprid (w/w) and 4.5% flumethrin (w/w). The imidacloprid component of the collar is aimed at the treatment and control of fleas and lice, and the flumethrin component at the treatment and control of ticks and mites. The medicated collar is expected to provide sustained protection over a period of eight months to dogs exposed to ticks, fleas and lice.

The objectives of the present studies are to demonstrate the efficacy of the imidacloprid/flumethrin collar formulation against fleas, ticks, mites and lice on dogs.

## Methods

All studies were conducted under the respective national animal protection legislation framework, and ethical approval was therefore obtained in all cases before study start.

### General materials and methods

Each study was designed to ascertain the immediate and/or long-term efficacy of a collar containing imidacloprid 10% (w/w) and flumethrin 4.5% (w/w) against a specific ectoparasite or ectoparasites on a group of experimentally infested dogs, compared to an untreated control group of dogs infested with the same ectoparasite or ectoparasites.

In accordance with the protocols for the various studies, custom-bred beagles or mixed-breed dogs that had not been treated with an acaricide, or insecticide, or a compound with an insect-growth regulating activity during the previous 60 to 90 days, were used. The dogs were maintained and handled with due regard for their welfare, and were acclimatized to the kennel environment seven to ten days prior to the commencement of a study. Dogs were individually housed in pens in indoor animal units that conformed to the national standards for floor area, lighting and temperature of the various countries in which the studies were conducted. Water was available *ad libitum* and an adequate amount of a commercial dog food towards their maintenance was provided daily.

Parent-stock fleas were reared on artificially infested dogs or cats, their eggs collected and incubated with flea rearing medium (dried, powdered beef blood and sand 1:4). The resultant adult fleas were collected and the numbers stipulated in the protocols for the various studies counted and placed in separate containers. These containers were opened on the dogs’ necks and the fleas were allowed to disperse in their hair. Parent-stock ticks were reared on artificially infested dogs or rabbits. Dogs were lightly sedated or restrained by hand and specified numbers of ticks were released onto their backs or necks and allowed sufficient time to disperse into the hair before the animals were released.

Approximately four days before the collars were fitted all animals were infested with a prescribed number of fleas or ticks of the species to be targeted. In some instances a slightly larger number of dogs than specified in the protocol were infested. One or two days after this infestation flea or tick counts were conducted. At this stage surplus animals that harboured the lowest numbers of fleas or ticks were excluded from the remainder of the study. The dogs that qualified for the study were ranked in descending order on their pre-treatment parasite counts, and animal ID’s were used to break ties. Thereafter they were randomly allocated to treatment groups. Each treated and each untreated control group of dogs consisted of at least seven individuals.

One or two days before the collars were fitted all animals in each study were infested with pre-determined numbers of fleas or of ticks or with both fleas and ticks of the species to be targeted. On Day 0 the test collars were fitted to the necks of the dogs allocated to the treatment group, and two days after the collars had been fitted flea and tick counts were done. The collar size was determined by the dog’s weight. Dogs that weighed less than 8 kg were fitted with collars designed for small dogs (35 cm in length) and those weighing more than 8 kg were fitted with collars designed for large dogs (66 cm in length). After the collar had been secured by means of the buckle the loose end was passed through the retaining loop or loops. The excess collar was cut off approximately 2–3 cm from the last retaining loop. At specified time intervals on the day of treatment all animals were carefully observed for adverse signs that could be ascribed to the collars or their active ingredients.

At prescribed intervals thereafter all the dogs in the study were re-infested with fleas or ticks depending on the target species. A day after these infestations flea counts were done and two days after the infestations tick counts were performed. This procedure was repeated at about 28-day intervals for approximately 34 weeks after the collars had initially been fitted.

Fleas were counted by combing the hair with a fine-toothed flea comb to recover fleas present in the animal’s pelage. Several strokes of the comb were made over each body region, each time moving in the same direction following the lie of the hair coat. Fleas showing no movement were considered dead. Tick counts were performed by intensive examination and palpation of the dog’s skin. When a tick was found the hair was parted and visual confirmation of the tick’s attachment status made. Once the ticks discovered by these methods had been removed, the dogs were either combed to ensure that all ticks had been collected, or thoroughly searched until the investigator felt confident that all ticks had been found. Male and female *R. sanguineus*, *D. reticulatus and D. variabilis* were counted, but only female *I. ricinus* and *I. scapularis* were counted because the males of *Ixodes* species seldom attach.

The experimental design of the various studies is schematically summarized in Table 1.

**Table 1:** Schematic Experimental Design of studies aimed at testing the efficacy of an imidacloprid 10.5%/flumethrin 4.5% collar against ectoparasites on dogs

**Study Day (Week)**	**Activity**
**−10 (approximately)**	Acclimatization to housing
**−7**	Pre-study flea or tick infestations
**−4**	Select dogs with highest flea or tick counts, and allocate them to treatment groups consisting of at least 7 untreated dogs and 7 treated dogs
**−2**	Tick infestation
**- 1**	Flea infestation
**0**	Fit collars to all dogs in treatment group
**2**	Flea and tick counts
**7 (=Week 1)**	Flea and/or tick infestation
**8, 9**	Flea counts Day 8, tick counts Day 9
**28 (=Week 4)**	Flea and/or tick infestation
**29, 30**	Flea counts Day 29, tick counts Day 30
**56 (=Week 8)**	Flea and/or tick infestation
**57, 58**	Flea counts Day 57, tick counts Day 58
**84 (=Week 12)**	Flea and/or tick infestation
**85, 86**	Flea counts Day 85, tick counts Day 86
**112 (=Week 16)**	Flea and/or tick infestation
**113, 114**	Flea counts Day113, tick counts Day 114
**140 (=Week 20)**	Flea and/or tick infestation
**141, 142**	Flea counts Day 142, tick counts Day 142
**168 (=Week 24)**	Flea and/or tick infestation
**169, 170**	Flea counts Day 169, tick counts Day 170
**196 (=Week 28)**	Flea and/or tick infestation
**197, 198**	Flea counts Day 197, tick counts Day 198
**224 (=Week 32)**	Flea and/or tick infestation
**225, 226**	Flea counts Day 225, tick counts Day 226
**238 (=Week 34)**	Flea and/or tick infestation
**239, 240**	Flea counts Day 239, tick counts Day 240

The studies were conducted at facilities in Germany, North America and in South Africa and the experimental designs of each were not necessarily identical. For the sake of uniformity, efficacy determinations have thus been allocated to 28-day or 4-week blocks, and any determination of efficacy during a 4-week window was included in the window within which it had been calculated.

Efficacy evaluations are in accord with those published in document EMEA/CVMP/EWP/005/2000-Rev.2 of the European Medicines Agency.

Percent efficacy against fleas was calculated as follows:

Efficacy $\left(\%\right)=100\text{ x }\left(\text{mc }–\text{ mt}\right)/\text{ mc}$, where

mc = geometric mean number of live fleas on the untreated control group of dogs

mt = geometric mean number of live fleas on the treated group of dogs.

The ticks collected from each dog were sorted into six categories according to their engorgement status and whether they were alive or dead (Table 2). Ticks showing no movement were considered dead.

**Table 2:** Classification of ticks on their state of attachment and engorgement and whether they are alive or dead

**Category**	**Condition**	**Attachment status**
**1**	Live	Unattached
**2**	Live	Attached, unengorged*
**3**	Live	Attached, engorged**
**4**	Dead	Unattached
**5**	Dead	Attached, unengorged*
**6**	Dead	Attached, engorged**

* no filling of the idiosoma evident.

** conspicuous filling of the idiosoma.

Acaricidal efficacy was calculated as follows:

Acaricidal efficacy$\left(\%\right)=100\text{ x}\left(\text{Gmc }–\text{ Gmt}\right)/\text{Gmc}$

Where Gmc = Geometric mean number of live ticks (categories 1–3) on dogs in the untreated control group at a specific time point.

Gmt = Geometric mean number of live ticks (categories 1–3) on dogs in the treated group at a specific time point

Sustained acaricidal efficacy against subsequent re-infestations, was calculated as follows:

Sustained efficacy $\left(\%\right)=100\text{ x}\left(\text{Gmc }–\text{ Gmt}\right)/\text{Gmc}$

Where Gmc = Geometric mean number of live ticks (categories 1–3) on dogs in the untreated control group at a specific time point.

Gmt = Geometric mean number of live and dead ticks (categories 1–3 & 6) on dogs in the treated group at a specific time point.

According to the EMEA guidelines, dead engorged ticks (Category 6) should be included with the 3 categories of live ticks as they had succeeded in engorging before being killed.

The use of geometric rather than arithmetic means for the calculation of efficacy in the studies reported here was justified by the statisticians involved in each study. Consequently, unless stated otherwise, all efficacy values have been based on a comparison of transformed geometric means of the individual parasite counts of treated and control animals. Geometric means better reflect the skewed distribution of the numbers of parasites recorded on individual animals within the small groups of animals of necessity included in an experimental study. In fact, in the case of anthelmintic efficacy studies, in which non-normal distributions of parasitic burdens are commonplace, the use of geometric means is practically a prerequisite. However, when large numbers of animals are involved, such as in field trials, both methods of calculating efficacy are acceptable.

Because zero flea and tick counts were recorded in many of the present studies, calculations were based on the geometric means of the individual flea or tick counts (count + 1). One (1) was subsequently subtracted from the result to obtain a meaningful value for the geometric mean of the study groups. Descriptive statistics (mean, minimum, maximum, standard deviation, CV%, geometric mean and median) on flea or tick counts for the various assessment days were also calculated.

## Fleas

### Efficacy against adult fleas

Four long-term studies, aimed at determining the efficacy of the imidacloprid/flumetrin collars against *C. felis felis*, were conducted. Infestation with fleas, the application of collars, the determination of flea numbers and the assessment of efficacy followed the procedures described in the General Materials and Methods and summarized in the Schematic Experimental Design (Table 1).

### Onset and speed of efficacy

In order to determine the onset of efficacy of the imidacloprid/flumetrin components of the medicated collars against *C. felis felis*, dogs were infested with fleas one day before the collars were fitted and efficacy was measured 24 h later. A second study was designed in an attempt to determine the time lapse between collar fitment and the elimination of fleas. In this study the control and the treated group of dogs in one of the long-term studies were infested with fleas on Day 6, after the collars had been fitted and fleas were collected 12 h later on Day 7. Once these collections had been completed the same two groups of dogs were infested on Day 7 and fleas collected 6 h later, and finally the same groups of dogs were again infested with fleas on Day 7 and fleas collected 2 h thereafter. Speed of efficacy could thus be determined at 2 h, 6 h and 12 h intervals after infestation.

### In vitro *larvicidal efficacy*

This study was designed to assess the effect that residues of imidacloprid and flumethrin on blankets that had been in contact with treated dogs, had on the development of flea larvae. The study commenced on Day-7 and continued until Week 35 after the collars had been fitted. Fleecy polyester blankets were fastened to wooden boards, which were then placed on the floor of transport boxes. The untreated control dogs and the collared dogs were confined in these boxes for 3 h on three consecutive days from Days −7 to −5 and again from Days 12 to 14 and thereafter for three consecutive days 3 days prior to each efficacy assessment day. At the end of each 3-day exposure period the blankets were removed and a circular sample, 8.5 cm in diameter, cut from the centre of each blanket. The wooden boards were cleaned with hot water, soap and ethanol and new blankets were attached before the next period of exposure to the control and treated dogs. The blanket samples were placed in Petri dishes and frozen at approximately −20°C for at least 24 h. After removal from the freezer the Petri dishes were allowed to reach room temperature. Thereafter, approximately 50 one-day old flea eggs were placed in the middle of each blanket-sample and about 0.5 g of flea rearing medium (dried, powdered beef blood and sand 1:4) was distributed in a very thin layer over the surface of each sample. The samples were incubated at 26 ± 2°C and 75 ± 8% relative humidity for 27 or 28 days. To facilitate counting of the adult fleas that had emerged, the Petri dishes were frozen at −20°C for at least 2 h. The geometric mean numbers of fleas on the blanket samples exposed to the untreated control dogs compared to the numbers on the blanket samples exposed to collared dogs were used to calculate efficacy.

### Flea allergy dermatitis

During the laboratory efficacy studies the dogs were repeatedly infested with a fairly large number of fleas, followed by a 4-week break. This is possibly the ideal way to provoke an allergic reaction. All the dogs in the various studies were observed daily and were clinically examined at pre-scribed times. Signs of flea allergy dermatitis and other conditions were recorded during these observations and examinations.

## Results and discussion

### Efficacy against adult fleas

Therapeutic efficacy against an existing population of *C. felis felis* varied between 99.8% and 100% on Day 2 after the collars had been fitted (Table 3). With the exception of one study, in which efficacy was 94.3% at 35 weeks after the collars had been fitted, sustained efficacy exceeded 95% in each of the 4-week study periods during the 35 weeks of the study.

**Table 3:** **Efficacy of imidacloprid 10%/flumethrin 4.5% collars against* Ctenocephalides felis felis*on dogs 24 h after each infestation**

**Study Day or Week**	**Activity and efficacy (%)**			
**Day −1 or −2**	**100 fleas**			
**Day 0**	Collars fitted to dogs in treated groups			
**Day 2** (therapeutic efficacy)	99.8	100	100	100
**Day 8**	100	100	–	–
**Week 4**	100	100	99.8	100
**Week 8**	99.7	99.3	98.9	100
**Week 12**	99.3	100	98.9	100
**Week 16**	98.5	100	98.4	99.3
**Week 20**	99.7	100	97.9	99.7
**Week 24**	99.2	100	98.2	99.9
**Week 28**	–	100	96.4	99.4
**Week 32**	–	99.7	96.6	96.2
**Week 35**	–	99.7	94.3	96.3

The sustained efficacy of the imidacloprid/flumethrin collars, without necessarily being flea-repellent in the strict sense of the word, implies that treated dogs will remain attractive to fleas, which will then be killed almost immediately, and certainly before they can lay eggs. Moreover, the existing flea population within a collared dog’s household or surroundings will progressively die-out as the animal will act as a lethal ‘vacuum-cleaner’ over a protracted period of time.

### Onset and speed of efficacy

Efficacy was 100% 24 h after the collars were fitted and was also 100% at 2 h, 6 h and 12 h after infestation on Day 6 or 7 after the collars had been fitted (Table 4).

**Table 4:** **The onset of efficacy of imidacloprid 10%/flumethrin 4.5% collars against adult *Ctenocephalides felis felis***

**Study Day**	**Activity**	**Efficacy (%)**
**Day −1**	100 fleas	
**Day 0**	Collars fitted	
**Day 1** (onset of efficacy)		100
**Day 6**	100 fleas	
**Day 7 (12 h after infestation on Day 6)**		100
**Day 7**	100 fleas	
**Day 7 (6 h after infestation)**		100
**Day 7**	100 fleas	
**Day 7 (2 h after infestation)**		100

The imidacloprid/flumethrin collars had an immediate lethal effect on an existing one-day old flea population, and also killed fleas within 2, 6 or 12 h after infestation. The results obtained in the onset of efficacy study were based on a single experiment, and the speed of efficacy results were obtained when the collars were 7 days old and the concentration of imidacloprid and flumethrin on the haircoat of the dogs presumably high. Further studies to support these findings are thus indicated. Nevertheless, the above results imply that under field conditions fleas that are present on a dog will be killed within 24 h of a collar being applied and that fleas in any new infestations acquired from the environment are likely to be killed within 2 h after getting onto a collared dog.

### In vitro *larvicidal efficacy*

The results of the study on the effect of imidacloprid and flumethrin residues on in-contact blankets on the larvae of *C. felis felis* are summarized in Table 5. The residues on blankets killed 99 to 100% of flea larvae on each assessment day during the 35 weeks of the study. This result can be interpolated to the treated dog’s home where it may utilize its bedding for sleeping or resting for several hours a day. Under these circumstances excellent sustained efficacy against flea larvae can be expected, thus resulting in a break in the flea’s life cycle and a reduction in the chances of the dog becoming infested.

**Table 5:** **Effect of imidacloprid/flumethrin residues on *Ctenocephalides felis felis *larvae on blankets in contact with collared dogs**

**Study Day or Week**	**50 flea eggs incubated on control and on in-contact blankets**
	**Larvicidal efficacy (%)**
**Day −7**	0
**Day 0**	Collars fitted
**Week 2**	100
**Week 5**	99
**Week 9**	100
**Week 14**	100
**Week 17**	100
**Week 21**	100
**Week 25**	100
**Week 29**	100
**Week 33**	100
**Week 35**	100

### Flea allergy dermatitis

Although the dogs in the long-term laboratory efficacy studies were repeatedly infested with a fairly large number of fleas, which on each occasion were removed and counted a day or two later, not one of the 51 collared dogs developed signs of flea allergy dermatitis (FAD), whereas 12 of the 51 untreated control animals developed signs suggestive of this condition (Table 6).

**Table 6:** Flea allergy dermatitis (FAD) in untreated control dogs compared to dogs wearing imidacloprid 10%/flumethrin 4.5% collars

**Duration of study**	**Number of control dogs**	**Number with FAD**	**Number of treated dogs**	**Number with FAD**
**24 weeks**	7	0	7	0
**33 weeks**	8	0	8	0
**33 weeks**	8	2	8	0
**35 weeks**	10	10	10	0
**35 weeks**	10	0	10	0
**35 weeks**	8	0	8	0
**Total**	**51**	**12**	**51**	**0**

Considering the multiple infestations with fleas during the long-term studies, it is perhaps surprising that so few dogs in the untreated control groups developed signs of FAD, and then only in two of the six long-term studies. This suggests that other factors may also have been involved in the latter two studies.

Since the clinical signs of FAD are directly related to exposure to flea bites, there are two main strategies in treating animals exhibiting this allergic reaction. The first is to reduce the animal’s exposure to flea bites and the second is to administer appropriate palliative treatment to reduce the pruritus and inflammation. A combination of both strategies usually produces the best results.

Spot-on products containing imidacloprid have been used for several years as an integral component in the treatment of FAD by eliminating the fleas responsible for the allergy. In a field trial conducted at several veterinary clinics in Italy, 1939 flea-infested dogs, of which more than 34% exhibited signs of FAD were presented for treatment [7]. A single treatment with a spot-on formulation of imidacloprid (Advantage®) caused a rapid decline in the percentage of dogs infested with fleas. At the same time there was a marked improvement in the frequency of pruritus associated with FAD until almost complete remission 28 days later [7]. There was also a marked improvement in the extent of alopecia during this time.

The effect of imidacloprid on fleas is rapid and in the present study the imidacloprid/flumetrin collars were 100% effective in killing fleas within 2 h of artificial infestation (Table 2). Thus fleas are likely to be eliminated before they can feed and provoke FAD by the injection of their saliva.

The use of ‘knock down’ insecticides, or those with only a limited time-span of efficacy, requires strict owner-compliance with the prescribed treatment regimen for dogs with FAD. However, the long-term efficacy of the slow-release imidacloprid component of the collars ensures sustained protection against flea bites, thus preventing intermittent exposure to flea saliva which initiates, provokes, sustains or exacerbates FAD.

### Ticks

## Methods

### Efficacy against adult ticks

In the studies described here, infestation with ticks, the application of the collars, the determination of tick numbers and the assessment of efficacy follow the procedures described in the General Materials and Methods and summarized in the Schematic Experimental Design (Table 1). Several long-term studies, during which dogs were repeatedly infested with ticks, were conducted in order to evaluate the efficacy of the collars against *R. sanguineus* (Table 7), *I. ricinus* and *I. scapularis* (Table 8), and against *D. reticulatus* and *D. variabilis* (Table 9).

**Table 7:** **Efficacy of imidacloprid 10%/flumethrin 4.5% collars against *Rhipicephalus sanguineus *on dogs 48 h after each infestation**

**Study Day or Week**	**Activity and efficacy (%)**			
**Day −2**	50 ticks (25♂ 25♀)	50 ticks (25♂ 25♀)	–	–
**Day −1**	–	–	50 ticks (25♂ 25♀)	50 ticks (both sexes)
**Day 0**	Collars fitted to dogs in treated groups			
**Day 2** (therapeutic efficacy)	84.8	64	46.5	32.2
**Day 7**	97	100	–	98
**Week 4**	100	100	98	100
**Week 8**	98.4	100	98.3	100
**Week 12**	99.7	99.6	100	99.1
**Week 16**	96.7	100	98.1	100
**Week 20**	98.5	100	99.4	100
**Week 24**	99.1	100	99.7	99.4
**Week 28**	–	100	96.9	96.6
**Week 32**	–	98.2	96.8	90.3
**Week 35**	–	100	99.1	93.2

**Table 8:** **Efficacy of imidacloprid 10%/flumethrin 4.5% collars against *Ixodes ricinus *and *Ixodes scapularis *on dogs 48 h after each infestation**

**Study Day or Week**	**Activity and efficacy (%)**		
**Tick species**	*Ixodes ricinus*		*Ixodes scapularis*
**Day −2**	40 ticks (20♂ 20♀)	40 ticks (20♂ 20♀)	–
**Day −1**	–	–	50 ticks (both sexes)
**Day 2** (therapeutic efficacy)	76.7	89.9	96.4
**Day 7**	100	100	100
**Week 4**	100	100	100
**Week 8**	100	100	100
**Week 12**	100	100	100
**Week 16**	100	100	100
**Week 20**	100	100	100
**Week 24**	100	100	100
**Week 28**	100	100	100
**Week 32**	100	100	100
**Week 34**	100	100	100

**Table 9:** **Efficacy of imidacloprid 10%/flumethrin 4.5% collars against *Dermacentor reticulatus *and *Dermacentor variabilis *on dogs 48 h after each infestation**

**Study Day or Week**	**Activity and efficacy (%)**		
**Tick species**	*Dermacentor reticulatus*		*Dermacentor variabilis*
**Day −2**	50 ticks (25♂ 25♀)	–	–
**Day −1**	–	50 ticks (25♂ 25♀)	50 ticks (both sexes)
**Day 0**	Collars fitted to treated group		
**Day 2** (therapeutic efficacy)	66.1	69.7	69.1
**Day 7**	99.5	–	–
**Week 2**	–	–	99.7
**Week 4**	100	99.1	–
**Week 8**	100	97.4	99.7
**Week 12**	99.7	98.7	98.5
**Week 16**	98.3	92.3	99.2
**Week 20**	97.2	97.5	94.4
**Week 24**	98.6	97.6	93.2
**Week 28**	–	98.5	92.6
**Week 32**	–	97.2	96.5
**Week 34**	–	96.4	90.1

### Onset and speed of efficacy

A single short-term study to determine the onset and also the speed of efficacy of the collars against *R. sanguineus* and *I. ricinus* was conducted. In this study collars were fitted to the treated group of dogs on Day 0 and directly thereafter all the dogs were infested with *R. sanguineus* and *I. ricinus*. Ticks were removed and counted 48 h later on Day 2. On the same day the dogs were re-infested with *R. sanguineus* and *I. ricinus* and efficacy determined 6 h later. The first reading represents the onset of efficacy against the ticks when treatment and infestation are almost simultaneous, and the second reading represents efficacy immediately thereafter, 6 h after infestation (Table 10).

**Table 10:** **Onset and speed of efficacy of imidacloprid 10%/flumethrin 4.5% collars against *Rhipicephalus sanguineus *and *Ixodes ricinus *on dogs**

**Study Day**	**Activity and efficacy (%)**	
**Tick species**	*R. sanguineus*	*I. ricinus*
**Day 0**	Collars + 50 ticks (25♂ 25♀)	Collars + 35 ticks (15♂ 20♀)
**Day 2** (onset of efficacy)	96.8	96.2
**Day 2**	Collars + 50 ticks (25♂ 25♀)	Collars + 35 ticks (15♂ 20♀)
**Day 2 (**6 h after infestation)	99.2	100

### Repellent efficacy

A single long-term study was conducted to test the repellent or rapid killing effect of the medicated collars against *R. sanguineus* and *I. ricinus*. In this study the dogs were infested with both tick species at 28-day intervals from Week 4 until Week 34 and repellent efficacy was evaluated 6 h after each infestation. At each occasion all the ticks on each dog were counted but not removed (Table 11). Other than this infestation with ticks, application of collars, and assessment of efficacy follow the procedures described in the General Materials and Methods and summarized in the Schematic Experimental Design (Table 1).

**Table 11:** **Repellent efficacy of imidacloprid 10%/flumethrin 4.5% collars against *Rhipicephalus sanguineus *and *Ixodes ricinus *on dogs 6 hours after each infestation**

**Study Day or Week**	**Activity and efficacy (%)**	
**Tick species**	*R. sanguineus* 50 ticks (25♂ 25♀)	*I. ricinus* 40 ticks (20♂ 20♀)
**Day 0**	Collars fitted to dogs in treated group	
**Week 4**	100	100
**Week 8**	99.7	100
**Week 12**	100	100
**Week 16**	99.4	100
**Week 20**	98.1	100
**Week 24**	99	100
**Week 28**	99	100
**Week 32**	97.6	100
**Week 34**	100	100

However, 48 h after each infestation all the ticks resulting from each infestation in the study were removed and counted. This was done because the first study was designed in such a manner that not only repellent effectiveness could be detected 6 h after each infestation, but also long-term sustained acaricidal efficacy could be determined 48 h after the same infestations (Tables 7, 8).

### In vitro *acaricidal efficacy*

This study was designed so that the *in vitro* effect that imidacloprid and flumethrin residues on the hair coat of a collared dog had on the nymphs and adults of *I. scapularis* could be measured. To this end three female dogs were acclimatized to the study conditions 10 days prior to treatment. Four days before treatment ten hair samples were taken from the same places on each animal, namely the dorsal and ventral surfaces, both lateral sides, and all four legs. The ten samples were first weighed and then combined and well-mixed for each dog separately. The total weight of the ten combined samples always exceeded 5.8 gm. Approximately 1 gm of hair from the combined sample of each dog was added to each of six Petri dishes. Ten unfed *I. scapularis* nymphs and ten unfed adults were added to the samples in the Petri dishes. These were counted two days later and all were still alive.

The three dogs were randomly allocated to three treatment groups and placed in separate pens. Imidacloprid/flumethrin collars were fitted to two of the dogs and the third remained untreated. One of the treated dogs was randomly chosen for hair-sampling and the other treated dog served as a backup for this dog.

Hair samples were collected from the designated treated dog and from the untreated dog 7 days and again 14 days after treatment and thereafter at 28-day intervals until Week 34. At every occasion the hair samples from each dog were placed in six separate Petri dishes lined with slightly moistened filter paper, and ten unfed *I. scapularis* nymphs and ten unfed adults were added to each dish, the lid of which was not secured with tape. Thereafter the Petri dishes were placed in an incubator tub containing a saturated salt solution to ensure a constant relative humidity. Approximately 48 h later the numbers of live ticks were counted in each Petri dish and efficacy determined (Table 12).

**Table 12:** ***In vitro *efficacy against *Ixodes scapularis *of residues of imidacloprid/flumethrin on the hair of a collared dog**

**Study Day or Week**	**Activity and efficacy (%)**	
	**10 unfed nymphs**	**10 unfed adult ticks**
**Day-2**	0	0
**Day 7**	100	100
**Day 14**	100	100
**Week 6**	100	100
**Week 10**	100	100
**Week 14**	98.7	100
**Week 18**	100	100
**Week 22**	100	100
**Week 26**	100	100
**Week 30**	100	100
**Week 34**	100	100

## Results and discussion

### Efficacy against adult ticks

At the commencement of the various studies the therapeutic efficacy of the collars against 1-day old *R. sanguineus* infestations varied between 32.2% and 46.5%, and against 2-day old infestations it varied between 64% and 84.8% (Table 7). Efficacy against re-infestation with ticks 7 days after the collars had been fitted exceeded 96%, and remained at this level or higher against re-infestations until the termination of one of the studies at 24 weeks, and in two studies terminated at 35 weeks. In the fourth study efficacy decreased to 90.3% by Week 32 and thereafter recovered to 93.2% by Week 35 (Table 7).

The therapeutic efficacy of the medicated collars against a 2-day old infestation of *I. ricinus* varied between 76.7% and 89.9%, and was 96.4% against a 1-day old infestation of *I scapularis* (Table 8). Thereafter the sustained acaricidal efficacy against re-infestations of either species remained at 100% for the 34-week duration of the study (Table 8).

The therapeutic efficacy of the collars was 69.7% against a 1-day old infestation of *D. reticulatus*, and 66.1% against a 2-day old infestation. The sustained acaricidal efficacy of the collars against re-infestation with *D. reticulatus* was consistently above 97% during the 24-week long study, and but for one transitory decline to 92% at 16 weeks, efficacy exceeded 96% in the 34-week long study (Table 9). The therapeutic efficacy of the collars against a 1-day old established infestation of *D. variabilis* was 69.1% and the repellent acaricidal efficacy against re-infestation at or above 98.5% during the initial 16 weeks of the study, and thereafter exceeded 90% until the termination of the study at 34 weeks (Table 9).

The collars were particularly effective against re-infestation with both *Ixodes* species that were targeted. Efficacy of 100% was recorded on every assessment day from Week 1 to Week 34 for *I. ricinus* and *I. scapularis*. Although the American tick, *D. variabilis* appeared to be slightly less sensitive to the flumethrin component of the collars than the European tick, *D. reticulatus*, efficacy against the former tick never dropped below 90% during the 34 weeks of the study.

As can be deduced from the results summarized in Tables 7, 8 and 9, the flumethrin component of the collars in many instances was not immediately >90% effective against ticks that had been attached to dogs for one or two days at the time-point of collar application (=therapeutic or curative efficacy). This was particularly noticeable for *R. sanguineus*. However, when infestation took place directly after the collars had been fitted (=preventive efficacy) effectiveness against both *R. sanguineus* and *I. ricinus* exceeded 96% (Table 10). Furthermore, two days after the collars had been fitted the concentration of flumethrin on the hair coat of collared dogs was already sufficient to reduce the number of surviving and/or feeding *R. sanguineus* and *I. ricinus* 6 h after infestation by 99.2% and 100% respectively (=repellent efficacy) (Table 10).

### Onset and speed of efficacy

Efficacy against *R. sanguineus* and *I. ricinus* applied immediately after the collars had been fitted exceeded 96%, and efficacy measured at 6 h after re-infestation on Day 2 exceeded 99% (Table 10). This implies that the majority of ticks that get onto a dog on the same day that the collars are fitted will be killed and that nearly every tick that gets onto a dog 2 days after the collars have been fitted will be eliminated within 6 hours (=repellent efficacy).

### Repellent efficacy

Repellent or’speed of kill’ efficacy measured 6 h after each infestation from Week 4 to Week 34 exceeded 97% against *R. sanguineus* and was 100% against *I. ricinus* for the duration of the study (Table 11). Taken in conjunction with the results obtained in the study in which the onset of efficacy was determined, repellent efficacy was already evident 2 days after the collars had been fitted (Table 10). Thus the vast majority of ticks that get onto a dog would be killed within 6 h from Day 2 after the collars had been fitted until at least 34 weeks thereafter.

A true repellent effect against ticks is evident when the ticks do not even attempt to attach and leave the host spontaneously. The perception of a repellent effect may instead be due to extremely rapid killing of ticks shortly after they have gained access to a host. The EMEA efficacy guidelines for testing of ectoparasiticides on dogs and cats allows for a 24 h time interval to claim a repellent effect, but in the studies described here, this interval was reduced to 6 h. The 6 h interval between infestation and the death of the majority of ticks would certainly interfere with the transmission of tick-borne pathogens, including *Ehrlichia* spp., which are normally transmitted soon after tick attachment [26].

### In vitro *acaricidal efficacy*

From Day 7 to Week 34 the efficacy of residues of imidacloprid/flumethrin on the hair coat of dogs against *I. scapularis* nymphs was 98.7% to 100%, and 100% against adult ticks (Table 12). Other *in vitro* studies using haircoat samples after fitting imidacloprid/flumethrin collars support these results, in that ataxia is evident 4 h after tick/hair contact, and nearly complete mortality is observed after approximately 12 h [27,28].

### Effect of shampooing or water immersion

It is inevitable that dogs with or without medicated collars are going to be washed or shampooed, or swim or go out into the rain. The former two events are usually planned, but the latter two can hardly be prevented by their owners. In all of these the question arises as to whether shampooing or getting wet will affect the efficacy of the medicated collars and whether the collars should be removed before washing or shampooing. The objective of the following study was to evaluate the efficacy of the imidacloprid/flumethrin collars against laboratory infestations of *C. felis felis*, *R. sanguineus* and *D. variabilis* on dogs that were either regularly shampooed or immersed in water.

Thirty-two dogs were allocated to four groups of eight dogs each based on sex and pre-treatment tick counts. Dogs in the first group served as untreated controls and those in the second group were fitted with imidacloprid/flumethrin collars, and both groups were shampooed regularly. Dogs in the third group served as untreated controls and those in the fourth group were fitted with medicated collars, and both of these groups were regularly immersed in water. The animals in all four groups were infested with *C. felis felis*, *R. sanguineus* and *D. variabilis*. The method of infestation, application of collars, determination of flea and tick numbers and assessment of efficacy followed the procedures described in the General Materials and Methods section and summarized in the Schematic Experimental Design (Table 1).

The initial infestations with *C. felis felis* and *R. sanguineus* took place on Day-1 and the collars were fitted one day later on Day 0. The initial infestations with *D. variabilis* only took place on Day 6. From Day 28 and onwards the dogs were infested with *R. sanguineus* seven days after they were infested with *D. variabilis*, which was followed one day later by *C. felis felis.* Flea counts were performed one day after infestation, and tick counts two days after infestation.

On Day 21 and at 28 day intervals thereafter all the dogs in the first two groups were shampooed with a shampoo not containing any active ectoparasiticidal ingredient. They were first wetted by means of a bath-shower and the shampoo was applied and the whole dog thoroughly lathered, followed by a thorough rinsing with approximately 19 litre of water applied over a period of 5 minutes by means of the bath-shower. On Day 21 and at 28-day intervals thereafter the dogs in Groups 3 and 4 were immersed in a tank containing tepid tap water for a minimum of 5 minutes and each animal’s head was thoroughly wetted three times during the procedure. On each day of this procedure the control group was immersed first followed by the collared group without changing the water. The collars were not removed from the dogs in either of the treated groups before shampooing or water immersion. After shampooing or immersion the dogs were allowed to air-dry in their runs. The tank was washed and filled with fresh tepid water before the next immersion procedure.

For the sake of legibility and conformity the infestations with fleas and ticks and concomitant efficacy evaluations have been aligned with each other as far as it is possible for each activity that took place during the same week after treatment.

## Results and discussion

The efficacy of the imidacloprid/flumethrin collars against fleas and ticks on dogs which had been shampooed or immersed in water is summarized in Table 13.

**Table 13:** Efficacy of Imidacloprid 10%/flumethrin 4.5% collars against fleas and ticks on dogs regularly shampooed or immersed in water

**Study Day or Week**	**Activity and efficacy (%)**					
**Flea or tick species**	*Ctenocephalides felis felis*		*Rhipicephalus sanguineus*		*Dermacentor variabilis*	
**Day-1**	100 fleas		50 ticks (mixed sexes)		N/A	
**Day 0**	Collars fitted to dogs in treated groups					
**Day 2**	100	100	55.8	83.1	N/A	
**Day 5**	–	–	–	–	50 ticks (mixed sexes)	
**Day 7**	100	100	98.4	97.8	99.5	97.8
**Treatment**	shampoo	immersion	shampoo	immersion	shampoo	immersion
**Day 21, Efficacy Day 28**	100	100	–	–	99.8	99.4
**Day 21 Efficacy Day 35**	–	–	100	99	–	–
**Day 49, Efficacy Day 56**	100	100	–	–	99.2	100
**Day 49 Efficacy Day 63**	–	–	99.7	100	–	–
**Day 77, Efficacy Day 84**	99.3	99.6	–	–	98	97.8
**Day 77, Efficay Day 91**	–	–	98.6	100	–	–
**Day 105, Efficacy Day 112**	97	96.3	–	–	97.8	97.3
**Day 105, Efficay Day 119**	–	–	100	100	–	–
**Day 133, Efficacy Day 140**	93.7	91.1	–	–	94.1	95.6
**Day 133, Efficay Day 147**	–	–	100	95.4	–	–
**Day 161, Efficacy Day 168**	98.3	87.1	–	–	95.9	95
**Day 161, Efficacy Day 175**	–	–	98.8	97.3	–	–
**Day 189, Efficacy Day 196**	93.9	67.8	–	–	95.4	96.1
**Day 189, Efficacy Day 203**	–	–	100	94.4	–	–
**Day 217, Efficacy Day 224**	90.1	50.3	–	–	94.7	89.1
**Day 217, Efficay Day 231**	–	–	97.6	94.4	–	–

From Day 28 and onwards dogs were infested one week later with *R. sanguineus* than they were infested with *C. felis felis* or *D. variabilis.*

= Dogs were shampooed or immersed in water one week before each re-infestation with *C. felis* and *D. variabilis* and this translated into two weeks before each re-infestation with *R. sanguineus.*

Efficacy against fleas remained above 90% throughout the study on the dogs that were shampooed, but efficacy declined to below 90% at 24 weeks after the initiation of the study in the group of dogs that were regularly immersed in water. Sustained acaricidal efficacy against re-infestation with *D. variablis* remained above 90% on dogs that were shampooed, but decreased to 89.1% at Week 32 on dogs that were immersed in water. Similarly sustained acaricidal efficacy against re-infestation with *R. sanguineus* remained above 95% throughout the study on the shampooed dogs, while it decreased to 94.4% at Week 28 on the dogs that had been immersed in water.

The results indicate that the acaricidal efficacy of the flumethrin component of the collars after shampooing or immersion in water was not affected to the same extent as the insecticidal efficacy of the imidacloprid component. This is probably because flumethrin is less water soluble than imidacloprid. It can also be assumed that the concentration of imidacloprid on the hair declined after each immersion in water and was then reloaded from the collar to a greater extent than flumethrin, thus resulting in an earlier depletion of the imidacloprid component in the collar. Strangely, however, thoroughly shampooing the dogs and then thoroughly rinsing them with water did not have the same negative effect on efficacy against *C. felis felis* as immersing them in water.

Similar studies have been conducted by Schuele et al. [29] in which the effect of shampooing or immersion in water on the efficacy of a spot-on formulation of pyriprole against *C. felis felis* and *R. sanguineus* was evaluated. In the one study efficacy against *C. felis felis* and *R. sanguineus* remained above 99% during the 30 days of the study. In the other efficacy against *C. felis felis* declined to 96% by Day 34 and to 93% against *R. sanguineus* by Day 24 in the group of dogs that were shampooed.

### Mites

## Methods

This study aimed to test the efficacy of imidacloprid/flumethrin collars against the mite *S. scabiei* on naturally infested dogs. Mixed breed dogs older than six months and judged to be naturally infested with *S. scabiei* were purchased from their owners. Dogs were included in the study provided that they harboured live *S. scabiei* mites and no live *Demodex* spp. mites, and that they had not been treated with an acaricide or an insecticide during the 8 weeks preceding Day 0 of the study. Ten dogs met these requirements.

Efficacy evaluation was based on the presence or absence of mites and on the improvement in skin lesions associated with sarcoptic mange. Because of the uncertainty factor in the mite count assessment, it was likely that false negatives could be recorded and this would result in an overestimation of the success rate of treatment. Consequently the success rate was defined as a dog that complied with all of the following conditions: no live mites, a complete resolution of the presence of papules and skin crusts and a > 90% improvement in body areas with hair loss by Day 90 after the collars had been fitted (Table 14).

**Table 14:** **Efficacy of imidacloprid 10%/flumethrin 4.5% collars against *Sarcoptes scabiei *on naturally infested dogs**

**Study Day**	**Observations and efficacy**			
**Criteria**	Success % (No. of dogs out of 10 positive for mites, papules and crusts and hair re-growth)			
**Component**	Mites	Papules	Crusts	Hair re-growth >90%
**Day −2**	0 (10/10)	10 (9/10)	10 (9/10)	100 (0/10)
**Day 0**	Collars fitted to all dogs			
**Day 29**	90 (1/10)	50 (5/10)	50 (5/10)	20 (2/10)
**Day 60**	90 (1/100	60 (4/10)	20 (8/10)	10 (1/10)
**Day 90**	100 (0/100	80 (2/10)	80 (2/10)	80 (8/10)

On Days −2, 29, 60 and 90, skin scrapings (+/− 4 cm²) were taken from five places on the dog’s body likely to be infested with mites, and the numbers of mites in these scrapings were counted. The clinical signs and extent of lesions on each dog were assessed on the days on which scrapings were made. The primary assessment variable in this study was the presence or absence of live mites on the dogs on each observation day following treatment. The presence or absence of live mites was then used to calculate a treatment success rate using the following formula:

Success rate (%) = No of dogs with no live mites/Total No. of dogs in group x 100

## Results and discussion

Mite counts were reduced to zero 90 days after application of the collars, and there was an 80% reduction in the number of dogs with papules or crusts as well as a > 90% improvement in hair re-growth in 80% of the dogs (Table 14).

Naturally infested dogs were chosen for the study because the skin lesions associated with artificial infestations can take weeks to develop [30], and in the process the dogs may be exposed to unnecessary discomfort. For the same reasons, no controls were kept, and for each dog the disappearance of mites and improvement in skin lesions served as an indication of treatment success.

Despite *S. scabiei* generally being susceptible to treatment [31,32] the lesions, and more particularly, the crusts associated with infestation, may only finally disappear weeks after the mites have been treated successfully. In a study by Fourie *et al*. [31] in which treatment resulted in a 100% reduction in mite counts, crusts were still present on two dogs 90 days after treatment. Although pruritus was observed in most of the dogs in the present study, its prevalence was not recorded because it is not considered to be a reliable indicator of the extent of infestation before treatment, or of efficacy after treatment. Some dogs with high mite counts might exhibit a low incidence of pruritus or none at all, while it may persist in dogs considered free of mites [32]. Because of the persistence of skin lesions and pruritus, it is essential that dog owners are informed not to expect an immediate improvement in their dog’s condition after treatment and that the process may take several weeks. At the same time, the dog owner should be informed that any recurrence of clinical signs associated with sarcoptic mange a month or more after treatment should be reported to the consulting veterinarian.

Sarcoptic mange is contagious and therefore treatment of an affected dog in a household in which there are several dogs should be accompanied by treatment of the other dogs, even though they may exhibit no clinical signs [24]. It is also advisable to warn the owner that the condition has temporary zoonotic implications for in-contact humans.

### Lice

## Methods

The objective of this study was to assess the efficacy of the imidacloprid/flumethrin medicated collars against natural infestations of the biting louse, *Trichodectes canis* on dogs. Eighteen of 26 dogs, naturally infested with *T. canis*, were enrolled in the study. These dogs were ranked from highest to lowest on total lice counts and randomly allocated to two groups, each consisting of nine dogs. All the dogs included in the study were infested with at least five lice. The lice were counted, without removing them, in eight locations on the dog’s body, namely both ears, the forehead, a square on the right and left sides of the thorax, a square on the abdomen, a strip along the midline of the back and on the base of the tail. The hair was gently separated and back-combed at each of these sites so that lice could be seen and counted. Light sedation was administered to facilitate the counting procedure.

On Day 0 imidacloprid/flumethrin collars were secured around the necks of the dogs assigned to the treated group. On Days 2, 7, 14, 21, 28 and 35, the numbers of lice in the eight locations on the body were counted. If the total lice count for a dog was zero, the ventral surface of the neck, the rump area of the hind leg, and the caudal aspect of each limb were searched. If no lice were detected in these areas, a general sweep of each side of the animal’s body was performed. If at any time the number of lice counted on a dog exceeded 100, the animal was combed and excess lice removed to prevent stress to the animal, but at least 10 adult lice, as well as nymphs and eggs were left on the dog.

Comparisons between the numbers of lice on the untreated control dogs and those of the collared dogs were used to calculate the efficacy of the collars against *T. canis*.

Efficacy $\left(\%\right)=100\times \left(\text{C}–\text{T}\right)/\text{C}$

C = Geometric mean numbers of *T. canis* on the untreated control group of dogs

T = Geometric mean numbers of *T. canis* on the treated group of dogs.

## Results and discussion

The efficacy of the imidacloprid/flumethrin collars against natural infestations of *T.* canis on dogs is summarized in Table 15. Efficacy against lice was 95.1% on Day 2 and 100% on Day 7 after treatment and remained at this level for the duration of the study. The dogs were thus completely cured from infestation with lice within 7 days of the collars being applied. Although the mean numbers of lice on dogs in the control group also decreased during the course of the study, the 100% efficacy of the collars from Day 7 onwards, meant that the decline in numbers of lice on the control group of dogs had no influence on efficacy.

**Table 15:** **Efficacy of imidacloprid 10%/flumethrin 4.5% collars against a natural infestation of *Trichodectes canis *on dogs**

**Study Day**	**Activity and efficacy (%)**
**No. of controls/No. of treated dogs**	9/9
**Day 0**	Collars fitted to dogs in treated group
**Day 2**	95.1
**Day 7**	100
**Day 14**	100
**Day 21**	100
**Day 28**	100
**Day 35**	100

Traditionally louse-infested animals are treated twice. The first treatment is aimed at the existing population of nymphs and adult lice, the second treatment, which is administered about one week later, is aimed at newly-hatched nymphs from eggs that had not been affected by the first treatment. The imidacloprid/flumethrin collars eliminate the necessity of a second treatment as any newly-hatched nymphs are immediately killed by the imidacloprid residues on the hair coat of the collared dogs.

## General discussion

The bench-mark of an insecticide to be used on dogs is its efficacy against *C. felis felis*, while the bench-mark for an acaricide is its efficacy against the predominant or most important tick species in the country or region in which it is to be used. Thus efficacy against *R. sanguineus* in regions with a mediterranean, tropical or subtropical climate is a must; efficacy against *I. ricinus*, and to an increasing degree *D. reticulatus*, in Europe with its somewhat cooler northern climate, is a prerequisite; and efficacy against *D. variabilis* and *I. scapularis* in the USA is essential. Efficacy against other flea or tick species must be regarded as a bonus, so too if efficacy includes ectoparasites belonging to other groups such as mites and lice. A broad-spectrum ectoparasiticide or combination of ectoparasiticides that is effective against the diverse assemblage of parasites encountered on dogs entrains a definite benefit to dog owners, as only a single treatment or application will be necessary. Should such an ectoparasiticide, or combination of ectoparasiticides also exhibit long-term efficacy against re-infestation, the benefit to owners and their pets is even more substantial. The imidacloprid/flumethrin collar would appear to meet all these criteria.

A number of other chemicals, either on their own or in combination with others, have also proved to have a sustained efficacy lasting for shorter or longer periods of time. Spot-on formulations of pyriprole as well as a combination of cyphenothrin and pyriproxyfen, have proved to be effective against fleas and ticks for 30 to 35 days after their application [29,33]. Efficacy against re-infestation with *R. sanguineus*, lasting for at least 170 days has been reported for a collar impregnated with a slow-release formulation of flumethrin and propoxur [34]. In contrast the efficacy of the imidacloprid/flumethrin collars against re-infestation with both fleas and ticks persists for at least 8 months.

Dogs that are not confined to kennels or indoors may harbour more than one group of ectoparasites, and within each group of ectoparasites infestation with two or more species is possible. Thus in northern Greece, France and Hungary several flea species were collected from dogs and a substantial number of these animals were simultaneously infested with more than one species [35-37]. In Albania 143 dogs infested with ectoparasites harboured three flea species, two tick species, two mite species and a biting louse, and mixed infestations were common [38]. In a kennel environment in South Africa dogs were infested with *Ctenocephalides* spp., and with *R. sanguineus*[39], and Matthee *et al*. [21] reported that a total of 28 tick species had been collected from dogs in various surveys conducted in that country. Although not recorded separately, several of the latter animals were simultaneously infested with as many as four tick species, while over a period of a year they may have harboured as many as nine species.

A sound knowledge of the seasonal abundance of fleas and ticks is crucial to determining the correct time to initiate chemical intervention. In both the Northern and Southern Hemisphere, infestation with fleas and ticks either commences, or escalates from a low base, during spring to reach peak numbers in mid or late summer. This peak is followed by a gradual or marked decrease in numbers towards winter. Thus at 13 veterinary practices in Hungary the proportion of dogs infested with fleas, of which the majority were *C. canis*, increased from 9.1% in spring to 18.1% and 19% in summer and autumn and declined to 9.9% in winter [37]. In Albania, the proportion of dogs infested with fleas, of which most were *C. canis*, increased from 64.3% in winter to 75.9% in spring and 100% in summer [38]. In South Africa infestation with *Ctenocephalides* spp., increased on dogs from spring to reach a peak in mid and late summer and declined thereafter to winter [39].

In Albania no *R. sanguineus* was detected on dogs in winter, whereas 34.2% of dogs were infested in spring and 50% in summer [38]. The largest numbers of *D. reticulatus* and *I. ricinus* were collected from dogs presented at 25 veterinary clinics in Hungary during March and April and in September, and the least in January and December [11]. The percentage of dogs, presented at veterinary practices in England, Scotland and Wales that were infested with *I. ricinus* and *Ixodes hexagonus* increased from March to reach a peak in June and declined thereafter, with a minor increase in October [14]. In Southeastern Georgia, USA, most adult *I. scapularis* were collected from dogs during the colder months from October to March, and most *D. variabilis* in the warmer months from April to August, and most *Amblyomma maculatum* in July and August [17]. In northern South Africa peak numbers of all developmental stages of *R. sanguineus* were recorded on dogs in a kennel environment from spring to late summer (October to April) [39], and a similar pattern of abundance for this tick was reported on dogs in the central region of this country [22].

The studies above confirm that flea infestation in both the Northern and Southern Hemisphere is generally most prevalent in the warmer months from spring to late summer. With the exception of *I. scapularis* in the USA, which apparently is a winter tick, the seasonality of tick infestations in both hemispheres generally mirrors that of fleas. It is thus logical that whatever mode of treatment is chosen it should commence in late winter or early spring and continue throughout the summer and into autumn. Usually this entails several treatments with parasiticides at monthly intervals throughout the activity period of the parasites. Furthermore, unless the chemical chosen is active against both fleas and ticks, multiple treatments with more than one chemical may be necessary to control both groups of parasites. The imidacloprid/flumethrin collar provides a sound alternative to a multi-treatment regimen for the control of fleas and ticks on dogs. If the collars are applied in late winter or early spring they will eliminate the existing small populations of fleas and ticks, and thereafter their sustained repellent efficacy will reduce the likelihood of re-infestation throughout the summer and into the autumn months.

The collars affect the flea and tick populations in different ways. *C. felis felis* can complete several generations during the year [8], and it is the extant population of fleas in spring that gives rise to their summer abundance. This cycle can be disrupted by applying the collars in spring and thus prevent re-infestation by successive populations. In addition the imidacloprid component of the collar that is shed on skin scales and hair of the treated dog will kill flea larvae in its bedding and resting places. Consequently the life cycle of *C. felis felis* will be interrupted during two critical phases. Firstly the egg-laying females are killed on the dog, and secondly the larvae, which will eventually give rise to fresh infestations with adult fleas, are eliminated in its immediate surroundings.

With the exception of *R. sanguineus*, which can complete two or more life cycles in a year on domestic dogs [39,40], most three-host ticks complete only a single life cycle annually. In the case of *R. sanguineus*, the immediate therapeutic efficacy of a newly applied collar will only affect some of the already attached ticks. However, the sustained acaricidal efficacy of the collar will prevent simultaneous re-infestation of the dog by larvae, nymphs and adults [41]. This will curtail the several annual life cycles completed by this tick in climates where the winters are mild and summers hot [39]. Because domestic dogs are for all practical purposes the only hosts of *R. sanguineus*, re-infestation of premises inhabited by a collared dog can only take place via an untreated dog. Even then any resulting re-infestation of the resident collared dog will be prevented by the long-term activity of the collar.

In the context of the other three-host ticks that infest dogs, their life cycles usually take a year to complete, and the immature stages of some of these tick species prefer rodents as hosts. Thus killing the adults on dogs during the summer will not only provide immediate relief to the dogs, but will also reduce the numbers of larvae and nymphs that are available to infest rodent hosts. This reduction of infestation on rodent hosts should translate into a lower level of infestation with adult ticks on dogs in the following year.

The adults of some three-host tick species have a wide range of hosts including dogs. In Europe these ticks include *D. reticulatus*, *I. ricinus*, *I. hexagonus*, in the USA *A. americanum*, *D. variabilis* and *I. scapularis*, and in southern Africa *Haemaphysalis elliptica* and *Rhipicephalus simus*. Thus although dogs may be protected from infestation within their immediate environment by the application of imidacloprid/flumethrin collars, they will be challenged by adult ticks of these species whenever they exercise with their owners, or are involved in hunting, or in any other outdoor activities. However, as demonstrated in the clinical trials above, they will be protected against this challenge by the sustained repellent efficacy of the collars, even if it is raining or if they go for a swim.

The immediate effect of the imidacloprid/flumethrin collar on an existing population of *C. felis felis* on dogs, and its sustained preventive efficacy, coupled with the larvicidal efficacy of imidacloprid residues in the dog’s immediate surroundings, result in interesting secondary benefits. One of these benefits is that the chances of a collared dog developing flea allergy dermatitis from repeated flea bites is significantly reduced. This stems from the fact that fleas that might re-infest the dog are killed within two hours, probably before they can start feeding. Moreover, Rust *et al.*[42] have demonstrated that cat hair treated with extremely small amounts of imidacloprid actually inhibits feeding by adult *C. felis felis*.

Other secondary benefits are at present speculative, but are likely to be thoroughly researched in the future. One of these is that the off-host lethal effect of imidacloprid against flea larvae precludes the possibility of the larvae ingesting the eggs of the cestode *D. caninum*. This will break the life cycle of the cestode prior to infection of the intermediate flea host. Even if fleas infected with the cysticercoids of *D. caninum* should get onto a collared dog, they in turn may be killed so rapidly that the dog would be unlikely to swallow one while nibbling at its skin.

The prevention of the transmission of tick-borne diseases by infected ticks, caused by the speedy repellent efficacy of the collars, is also still speculative. However, because efficacy is already evident within 6 h of re-infestation it would seem unlikely that infected ticks would have had time to transmit whatever organisms with which they may be infected to the collared dog. Should a dog already be infected with a tick-borne disease, application of an imidacloprid/flumethrin collar will not affect the course of the disease as the tick vector will most probably already have detached. However, application of the collar at this time may protect the dog against re-infection by eradicating other ticks infected with the same organism should they climb on to it. If there are other dogs in the same household as the sick dog, they must be fitted with collars. This will assist in protecting them against infestation with ticks that may be infected with the same pathogen.

Shaw *et al*. [2] have listed the various tick-borne diseases that affect dogs in different regions of the world and also the ticks responsible for transmitting the causative organisms of these diseases. Not surprisingly, *R. sanguineus* is involved in the transmission of several of them, namely *Babesia canisBabesia vogeliBabesia gibsoniHepatozoon canisEhrlichia canis* and *Rickettsia conorii*. *D reticulatus* is responsible for the transmission of *B. canis* in Europe and *D. variabilis* for the transmission of *Ehrlichia chaffeensis* and *Rickettsia rickettsii* in the USA [2]. Recent studies indicate that the distribution range of *D. reticulatus* in several European countries as well as in Britain is expanding [9,15,28]. This has led to cases of canine babesiosis being detected in regions where the disease had previously not been encountered [28]. In the light of these findings treatment of dogs for ticks is even more imperative. Treatment that kills ticks and thus reduces the effective population that can transmit disease is essential. If, however, it can be proved that the rapid killing of infected ticks on dogs within six hours of infestation could actually prevent transmission of disease, this would be a huge step forward.

## Conclusion

The rapid insecticidal and acaricidal properties of the medicated collars against newly-acquired infestations of fleas and ticks and their sustained high levels of preventive efficacy have been clearly shown. Consequently they have the potential to prevent the transmission of vector-borne diseases and other conditions directly associated with infestation throughout an entire season of parasite abundance.

## Competing interests

These clinical studies were completely funded by Bayer Animal Health GmbH, Monheim, Germany, of which D. Stanneck (Germany) and K. Krieger are employees, and by Bayer HealthCare LLC, Animal Health (USA). ClinVet is an independent Contract Development Organisation, which was contracted to manage the conduct of a part of these studies. I.G. Horak is a long-term, contract employee of Clinvet and an Honorary Professor at the Universities of the Free State and Pretoria. All authors voluntarily publish this article and have no personal interest in these studies other than publishing the scientific findings that they have been involved in via planning, setting-up, monitoring and conducting the investigations and analysing the results.
